# Double-carbapenem therapy in the treatment of multidrug resistant Gram-negative bacterial infections: a systematic review and meta-analysis

**DOI:** 10.1186/s12879-020-05133-0

**Published:** 2020-06-11

**Authors:** Yuan-yuan Li, Jin Wang, Rui Wang, Yun Cai

**Affiliations:** 1 Center of Medicine Clinical Research, Department of Pharmacy, PLA General Hospital, 28 Fu Xing Road, Beijing, 100853 People’s Republic of China; 2 PLA Medical School, Beijing, China

**Keywords:** Double-carbapenem therapy, Antibiotics, Carbapenem-resistant Enterobacteriaceae, Multidrug resistant, Meta-analysis

## Abstract

**Background:**

To compare the efficacy and safety of double-carbapenem therapy (DCT) with other antibiotics for the treatment of multidrug resistant (MDR) Gram-negative bacterial infections.

**Methods:**

Cochrane Library, PubMed, Embase and Web of Science as well as Chinese databases were searched from database establishment to February 2019. All types of studies were included if they had evaluated efficacy and safety of DCT regimens in patients with MDR Gram-negative bacterial infections. Clinical response, microbiological response, adverse events and mortality were the main outcomes. The protocol was registered with PROSPERO No. CRD42019129979.

**Results:**

Three cohort or case-control studies consisting of 235 patients and 18 case series or case reports consisting of 90 patients were included. The clinical and microbiological responses were similar between DCT and other regimens in patients with carbapenem-resistant Enterobacteriaceae (CRE) infection. DCT achieved a lower mortality than comparators in patients with CRE infection (OR = 0.44, 95% CI = 0.24–0.82, *P* = 0.009). Ertapenem was the most reported antibiotic in DCT regimens in case series or case reports. Moreover, clinical and microbiological improvements were found in 59 (65.6%) and 63 (70%) in total 90 cases, respectively.

**Conclusions:**

DCT was as effective as other antibiotics in treating MDR Gram-negative bacterial infections, with similar efficacy response and lower mortality. DCT could be an alternative therapeutic option in the treatment of MDR Gram-negative bacterial infections. High-quality randomized controlled trials were required to confirm the beneficial effects of DCT.

## Background

Carbapenem antibiotics (including imipenem, meropenem, ertapenem and doripenem), with a broad spectrum of antibacterial activity, play an extremely important role in the field of anti-infective treatment for severe infections. They are stable against most chromosomal broad-spectrum beta-lactamases and cephalosporinases found in Gram-negative bacteria [1, 2]. However, with the wide application of carbapenem antibiotics, carbapenem-hydrolyzing beta-lactamases, also named carbapenemases, have been increasingly found in Gram-negative pathogens. These beta-lactamases may limit the use of carbapenem antibiotics and cause treatment failure in severe infections [3, 4]. Carbapenemases, accompanied with drug resistance, constantly threat global health [5].

Carbapenemases belong to Ambler class A, B or D beta-lactamases and are mostly produced by Enterobacteriaceae, *Pseudomonas aeruginosa* or *Acinetobacter baumannii* [6, 7]. Class A carbapenemases can effectively hydrolyze carbapenem antibiotics by binding on active-site serine. These carbapenemases include the members of SME (*Serratia marcescens* enzyme), NMC (non-metallo enzyme carbapenemase), IMI (imipenem-hydrolyzing), GES (Guiana extended spectrum) and the most important KPC (*Klebsiella pneumoniae* carbapenemase) beta-lactamases [8]. Class B carbapenemases are also called Metallo-beta-lactamases (MBLs). These zinc-dependent enzymes can hydrolyze beta-lactams and are not inhibited by beta-lactamase inhibitors. Class B carbapenemases include IMP (imipenemase), VIM (Verona integron-encoded MBL), SPM (Sao Paulo MBL), GIM (German imipenemase) and NDM (New Delhi MBL) groups [9, 10]. They are mainly detected in *P. aeruginosa* and Enterobacteriaceae [11]. Class D carbapenemases are primarily detected in *A. baumannii* and Enterobacteriaceae (especially *K. pneumoniae*). They consist of oxacillinases (OXAs) which prefer to hydrolyze oxacillin or cloxacillin at higher rates than penicillin. Most members of OXAs are not susceptible to beta-lactamase inhibitors, but may be inhibited by NaCl [12, 13].

For carbapenem-resistant Gram-negative bacteria, there are limited antimicrobial treatment options [14, 15]. Novel beta-lactam/beta-lactamase inhibitors (such as ceftazidime/avibactam) may be available treatment options, while poor use and rapid emergence of resistance restrict their application [16, 17]. With less effective monotherapy and increasing resistance, evidence of retrospective studies on combination therapy of Gram-negative bacterial infections is increasing [18–20]. Many combination therapies have shown better survival and mortality reduction compared with monotherapy regimens, especially patients with a high predicted mortality [21–23]. The carbapenem-based combination regimens exert good synergistic results and low resistance [24, 25]. In these combination regimens, double-carbapenem therapy (DCT) is first attempted in three Greek patients in 2013 [26]. This study demonstrated bactericidal effect and clinical success of DCT and attributed to inactivating carbapenemases made by one carbapenem, mainly ertapenem. Since then, more and more clinical studies about DCT have been reported, while its effectiveness and safety have not been comprehensively addressed. In the present systematic review and meta-analysis, we aimed to evaluate efficacy and safety of DCT and other antibiotic regimens in patients with multidrug resistant (MDR) Gram-negative bacterial infections.

## Methods

This meta-analysis followed the Preferred Reporting Items for Systematic Reviews and Meta-analysis (PRISMA) guidelines [27] and was registered to PROSPERO (No. CRD42019129979) [28].

### Search method and data extraction

Literature search was performed in English databases, including Cochrane Library, PubMed, Embase and Web of Science, and Chinese databases, including SinoMed, CNKI and WANFANG MED DATA, from database establishment to February 3rd, 2019. No restrictions on language and geographic region were applied. Screening of abstract and full text was independently performed by two authors (YYL and JW). Search terms were set as “Double carbapenem”, “Dual carbapenem”, “Carbapenem” AND “Double”, “Carbapenem” AND “Dual”, “Carbapenem” AND “Joint”, “Carbapenem” AND “Combination”. Reference lists of included articles and relevant reviews were also searched.

In order to ensure accuracy, data extraction was independently carried out by two authors (YYL and JW). Controversial issues were resolved by consensus. When necessary, the corresponding authors were requested to provide unpublished data via e-mail. The following information was extracted: first author name, publication year, region, study design, characteristics of patients (sample size, sex and age), type of infection, type of organism, administered antibiotics, antimicrobial susceptibility test, treatment duration, follow-up time and outcomes.

### Inclusion and exclusion criteria

Randomized controlled trials (RCTs), cohort and case-control studies as well as case series and case reports were included. Studies focusing on patients with MDR Gram-negative bacterial infections were considered eligible for the meta-analysis, if such studies had reported available data of clinical response, microbiological response, adverse events, or mortality for the treatment of DCT. Studies which contained DCT in both treatment and control groups were excluded. Studies on experimental animal models and in vitro studies were excluded.

### Quality assessment

Regarding risk of bias, the quality of each selected RCT was independently assessed by the two authors with the Cochrane Collaboration’s tool [29]. The non-randomized studies were assessed with the Newcastle-Ottawa Scale (NOS) [30]. The risk of bias was evaluated as low, median or high by assigning or scoring each item separately. Any differences were resolved through consensus.

### Definitions and outcomes

The definitions of infections in the current meta-analysis were based on the definitions provided by the individual studies. The outcomes mainly focused on the efficacy and safety of DCT, including clinical response, microbiological response, adverse events and mortality. Clinical response was defined as resolution of clinical signs and symptoms of the infections by therapy completion. Microbiological response was defined as the absence of pathogens from subsequent specimen cultures.

### Statistical analysis

Meta-analysis was performed by Review Manager 5.3. Odds ratios (ORs) were calculated as effect measures, and *P* < 0.05 was considered as statistically significant. The fixed effects model was used to obtain pooled estimates of ORs, including 95% confidence interval (CI) [31]. Statistical heterogeneity was tested by □^2^ test (*P* ≤ 0.10 to indicate statistically significant) and quantified using I^2^ statistics [32]. Subgroup and sensitivity analyses were performed according to treatment regimens in control groups. The publication bias was assessed through visual inspection of funnel plot.

## Results

### Study identification

**Fig. 1:**
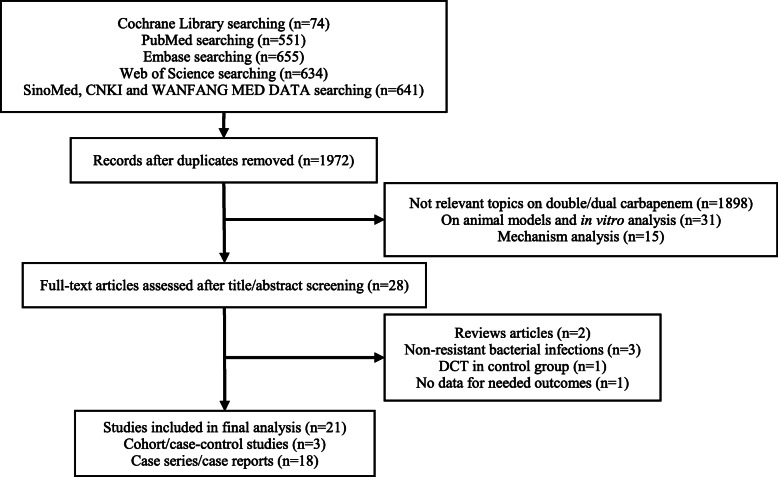
Flowchart of study selection. Abbreviations: DCT: double-carbapenem therapy

A total of seven databases and 1972 unique references were initially identified. Overall, 28 studies were selected for full-text review, and 21 studies met our inclusion criteria. For these 21 studies, three trials [33–35] were cohort or case-control studies, and 18 reports [26, 36–52] were case series or case reports. The flow diagram (Fig. 1) showed the detailed screening and selection process for the trials included in our analysis. Table 1 summarized the basic characteristics of cohort or case-control studies included, and such information for case series or case reports was performed in Table 2. Our study covered patients with MDR Gram-negative bacterial infections, and DCT was used to compare with other available antibiotics.

**Table 1:** Basic characteristics of cohort/case-control studies included

Author/Year	Region	Design	Infection	Organism	Sample size (D/C)	Age^a^ (years) (D/C)	Antibiotics		Antimicrobial susceptibility test (μg/ml) (D/C)	Treatment duration^a^ (days) (D/C)	Follow-up time (days) (D/C)	Outcomes^b^
							DCT	Control				
Pascale, 2017 [33]	Italy	retrospective study	VAP (51), HAP (23), BSI (16), SBSI (49), CVCI (18), UTI (12), IAI (19), SSTI (12), MSI (7)	CRKP	48/96	D: 55.5 ± 15 C: 61.3 ± 12	ETP 1 g q12h/2 g q24h MEM 2 g q8h (3 h infusions)	CST GEN TGC monotherapy or combined	CAB ≥1 R 48/96 CST ≤2 S 28/64 GEN ≤2 S 15/72 TGC ≤1 S 16/58	D: 17 (11.5–25.5) C: 11.5 (7.5–15.5)	90/90	①②③
Venugopalan, 2017 [34]	USA	retrospective study	BSI	CRKP	18/18	D: 72(61–83) C: 62(48–75)	ETP 1 g q24h DOR 2 g q8h (4 h infusions)	DOR + CST	ETP - DOR^c^ 8 (8–32)/− CST^c^ 8 (0.5–12)/1.25 (0.75–3.5)	D: 12 (7–14) C: 9 (7–12)	30/30	①②③
Cancelli, 2018 [35]	Italy	retrospective study	PNA (12), BSI (14), UTI (37), STI (12)	CPCRE	21/34	D: 62.28 ± 12.1 C: 61.18 ± 17	ETP 1 g/d MEM 6 g/d	CST CST + TGC/GEN/RIF/CAB AMG AMG + CAB MEM + FLQ	ETP^d^ 256 R 20/− ^e^ MEM^d^ 256 R 20/− ^e^ CST R 10/6 TGC R 11/18 RIF - AMG R 8/4 FLQ R 21/31	D: 39.2 ± 29.5 C: 20.4 ± 14.1	60/60	①③

Abbreviations: *D* double-carbapenem therapy (DCT) group, *C* the control group, *PNA* pneumonia, *HAP* hospital-acquired pneumonia, *VAP* ventilator-associated pneumonia, *BSI* bloodstream infection, *SBSI* secondary bloodstream infection, *CVCI* central venous catheter infection, *UTI* urinary tract infection, *IAI* intra-abdominal infection, *SSTI* skin and soft tissue infection, *STI* soft tissue infection, *MSI* multiple site infection, *CRKP* carbapenem-resistant *K. pneumoniae*, *CPCRE* carbapenemase producing carbapenem-resistant Enterobacteriaceae, *MEM* meropenem, *ETP* ertapenem, *DOR* doripenem, *GEN* gentamicin, *CST* colistin, *TGC* tigecycline, *RIF* rifampicin, *AMG* aminoglycosides, *CAB* carbapenem antibiotics, *FLQ* fluoroquinolones, *S* sensitive, *R* resistant

-: not reported

^a^. Data are expressed as mean ± standard deviation (SD), or median (range or interquartile range)

^b^. ①clinical response; ②microbiological response; ③mortality

^c^. The minimal inhibitory concentration (MIC) of antibiotics is expressed as mean or median (interquartile range)

^d^. The MIC of antibiotics is represented by MIC50

^e^. The remaining strains are not available

**Table 2:** Basic characteristics of case series/case reports included

Author/ Year	Region	Design	Sample size	Sex	Age^a^ (years)	Infection	Organism	DCT	Combined antibiotics	Antimicrobial susceptibility test (μg/ml)	Treatment duration^a^ (days)	Follow-up time^a^ (days)	Outcomes
Ceccarelli, 2013 [51]	Italy	case report	1	male	65	SBSI, VAP combined	MDR, KPC-III-producing KP	ETP 0.5/1 g q24h DOR 0.25/0.5/1 g q8h (4 h infusions)	–	ETP ≥8 R DOR R	28	30	clinical response microbiological response
Giamarellou, 2013 [26]	Greece	case report	3	male (1) female (2)	54 42 44	SBSI (2), UTI (3)	PDR, KPC-II-producing KP	ETP 1 g q24h MEM 1/2 g q8h (2) DOR 2 g q8h (1)	–	ETP > 8 R MEM > 16 R DOR > 8 R	20 14 10	300 21 180	clinical response 3/3 microbiological response 3/3
Oliva, 2014 [38]	Italy	case report	3	male (3)	–	API (1), BSI (2)	PDR, CPKP	ETP 0.5/1 g q24h MEM 1 g q12h/2 g q8h	–	ETP 128 R (1), 256 R (2) MEM 128 R (1), 256 R (2)	21 2 24	–	clinical response 3/3 microbiological response 3/3 demised 1/3
Camargo, 2015 [52]	USA	case report	1	female	64	IAI, VAP, BSI combined	XDR, KPC-producing KP	ETP 1 g q24h MEM 1 g q12h	CST	ETP - MEM - CST 12 R	12	–	microbiological failure emergence of colistin resistance switched to AVC + ETP
Chua, 2015 [41]	Singapore	case report	2	male (2)	62 77	SSI (1), HAP (1)	KPC-producing KP	ETP 0.5/1 g q24h DOR 0.5/1 g q8h (4 h infusions)	PMB + CST PMB	ETP 4 (1), > 32 (1) DOR 8 (1), − (1) PMB 1 (1), − (1) CST -	12 10 + 7	30 13	clinical response 2/2 microbiological response 2/2 demised 2/2
Oliva, 2015 [37]	Italy	case report	1	female	75	CVCI	PDR, KPC-producing KP	ETP 1 g q24h MEM 2 g q8h	CST	ETP 128 R MEM 256 R CST 32 R	21	–	clinical response microbiological response
Tumbarello, 2015 [50]	Italy	case report	8	–	≥18	BSI	KPC-producing KP	ETP MEM	–	ETP - MEM -	≥2	14	demised 3/8
Alessandra, 2016 [46]	Italy	case series	15	male (10) female (5)	60.9 ± 10.9	UTI (8), SSTI (2), EPI (2), PNA (1), MSI (2)	KPC-producing KP	ETP 1 g (1 h infusions) MEM 2 g q8h (3 h infusions)	–	ETP > 8 R (14) MEM > 16 R (14) ETP, MEM > 32 R (1)	15 (7–150)	60	clinical response 12/15 microbiological response 12/15 adverse events 3/15 (nausea, hypernatremia and seizures) demised 1/15
Cprek, 2016 [47]	USA	case series	18	male (10) female (8)	62.5(51–67)	BSI (7), PNA (5), IAI(2), UTI (3), SSSI (1)	CRKP	ETP 1 g q24h MEM 2 g q8h (17) DOR 0.5 g q8h (1)	DOX, GEN AMK, CIP TGC + PMB CIP + TGC GEN + DOX	CAB > 1 R DOX, GEN, AMK, CIP, TGC, PMB -	17 (2–72)	30	clinical response 7/18 microbiological response 11/14 adverse events 2/18 (2 seizures) demised 5/18
Montelione, 2016 [40]	Italy	case report	1	male	62	API	XDR, CPKP	ETP 1 g q24h MEM 2 g q8h	–	ETP 128 R MEM 256 R	28	1095	clinical response microbiological response
Oliva, 2016 [39]	Italy	case report	1	female	61	SSI, HAP, SBSI combined	KPC-producing EC	ETP 0.5 g q24h (1 h infusions) MEM 2 g q12h (3 h infusions)	–	ETP 16 R MEM 32 R	10	–	clinical response microbiological responsedemised
Basaranoglu,2017 [44]	Turkey	case report	3	male (2) female (1)	3 months 8 months 3	SBSI (1), CRBSI (2)	MDR KP	ETP 0.015 g/kg q12h MEM 0.02–0.04 g/kg q8h	CIP + TGC AMK + TGC + CIP CIP + AMK + CST	ETP > 8 R (1), > 32 R (2) MEM > 6 R (1), > 32 R (2) CIP > 2 R (1), > 4 R (2) TGC 1 S (1), 2 S (1), > 2 R (1) AMK 16 IR (1), > 64 R (2) CST -	14 15 26	–	clinical response 2/3 microbiological response 3/3
Nekidy, 2017 [36]	United Arab Emirates	case report	1	female	62	SSI, UTI, PNA, BSI combined	MDR KP	ETP 1 g q24h MEM 1 g q8h	–	ETP ≥8 R MEM -	28 + 7, 10, 14, 28, 14	–	clinical response microbiological response
Souli, 2017 [45]	Greece	case series	27	male (15) female (12)	59(15–83)	BSI (13), UTI (12), VAP (1), EVDI (1)	PDR/XDR, KPC-II-producing KP	ETP 1 g q24h (1 h infusions) MEM 2 g q8h (3 h infusions)	–	ETP > 8 IR MEM ≥2 IR	10 (5–28)	28 (9–200)	clinical response 21/27 microbiological response 20/27 adverse events 4/27 (generalized rash, eosinophilia and 2 aseptic meningitis) demised 8/27
Carrasco,2018 [42]	Spain	case report	1	female	36	BSI	XDR, KPC-producing KP	ETP 1 g q24h MEM 2 g q8h (3 h infusions)	–	ETP ≥32 R MEM ≥32 R	14	90	clinical response microbiological response
Galvão, 2018 [43]	Brazil	case report	1	male	59	SSI, SBSI combined	XDR, KPC-producing KP	ETP 1 g q24h MEM 2 g q8h (4 h infusions)	AMK + LZD +FCA	ETP ≥8 R MEM ≥16 R AMK 4 S LZD - FCA -	45	–	multiple organ failure and demised
Liang, 2018 [48]	China	case report	1	male	50	SBSI	XDR KP	ETP 1 g q24h (1 h infusions) MEM 1 g q8h (3 h infusions)	–	ETP R MEM R	9	–	clinical response microbiological response
Rosa, 2018 [49]	USA	case report	2	male (1) female (1)	57 35	UTI	NDM-harboring KP/EC	ETP 1 g q24h MEM 1 g q12h (4 h infusions)	FOF	ETP - MEM ≥16 R FOF 12 S (1), 256 R (1)	14	–	clinical response 2/2 microbiological response 2/2

Abbreviations: *DCT* double-carbapenem therapy, *PNA* pneumonia, *HAP* hospital-acquired pneumonia, *VAP* ventilator-associated pneumonia, *BSI* bloodstream infection, *SBSI* secondary bloodstream infection, *EPI* endovascular prosthesis infection, *API* Aortic periprosthetic infection, *CVCI* central venous catheter infection, *CRBSI* catheter-related bloodstream infection, *UTI* urinary tract infection, *IAI* intra-abdominal infection, *SSTI* skin and soft tissue infection, *SSSI* skin and skin structure infection, *SSI* surgical site infection, *EVDI* external ventricular drainage infection, *MSI* multiple site infection, *KP Klebsiella pneumoniae*, *EC Escherichia coli*, *CRKP* carbapenem-resistant *K. pneumoniae*, *CPKP* carbapenemase-producing *K. pneumoniae*, *KPC K. pneumoniae* carbapenemase, *KPC-II* a type II carbapenem against KPC-producers, *KPC-III* a type III carbapenem against KPC-producers, *NDM* New Delhi Metallo-beta-lactamase, *MDR* multidrug resistant, *XDR* extensively drug resistant, *PDR* pandrug resistant, *MEM* meropenem, *ETP* ertapenem, *DOR* doripenem, *CAB* carbapenem antibiotics, *CST* colistin, *GEN* gentamicin, *TGC* tigecycline, *CIP* ciprofloxacin, *AMK* amikacin, *FOF* fosfomycin, *LZD* linezolid, *PMB* polymyxin B, *DOX* doxycycline, *FCA* fluconazole, *AVC* ceftazidime/avibactam, *S* sensitive, *I* intermediate, *R* resistant

-: not reported

^a^. Data are expressed as mean ± standard deviation (SD), or median (range or interquartile range)

The three cohort or case-control studies were composed of 235 patients with carbapenem-resistant Enterobacteriaceae (CRE) infection. Colistin, tigecycline and aminoglycoside (especially gentamicin) monotherapies or combined regimens were compared with DCT in all patients. DCT regimens included ertapenem+meropenem and ertapenem+doripenem. Ertapenem was used at a daily dose of 1–2 g. Meropenem and doripenem were administered every 8 h at a high dose (2 g), mainly adopting the extended infusion. Dose was adjusted according to creatinine clearance if renal function was abnormal.

### Study quality

The NOS assessment tool included three subjects as follows: the selection of study groups, the comparability between the groups and the ascertainment of exposure or outcome. Studies with a score of 7–9 were considered as high-quality studies [53, 54]. Table 3 summarized the risk of bias. All studies in our meta-analysis had high qualities (7 score) and low risk for sequence generation and allocation concealment.

**Table 3:** Risk of bias assessed by NOS assessment tool

Author/Year	Design	Selection	Comparability	Outcome /Exposure	NOS score
Pascale, 2017	case-control study	⁕⁕⁕	⁕⁕	⁕⁕	7
Venugopalan, 2017	cohort study	⁕⁕⁕⁕	⁕⁕	⁕	7
Cancelli, 2018	cohort study	⁕⁕⁕⁕	⁕⁕	⁕	7

### Clinical response

**Fig. 2:**
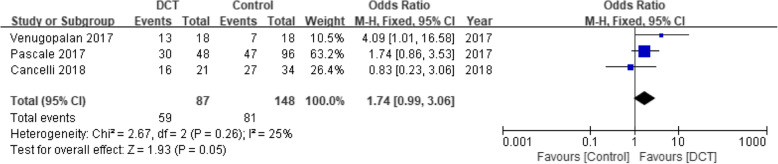
Forest plot of clinical response for patients with CRE infection. Abbreviations: CRE: carbapenem-resistant Enterobacteriaceae; CI: confidence interval

The data pooling from three studies consisting of 235 patients reported no significant difference between DCT-treated patients and those treated with other antibiotics (OR = 1.74, 95% CI = 0.99–3.06, *P* = 0.05) (Fig. 2).

### Microbiological response

**Fig. 3:**
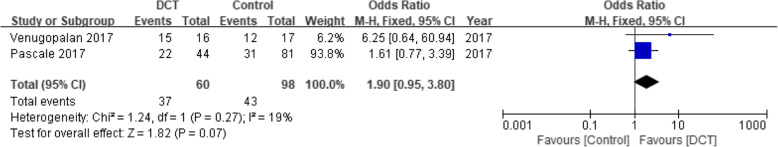
Forest plot of microbiological response for patients with CRE infection

Two studies consisting of 158 patients had reported the microbiological response. No significant difference was detected in patients with CRE infection between DCT and control groups (OR = 1.90, 95% CI = 0.95–3.80, *P =* 0.07) (Fig. 3).

### Adverse events

No studies had recorded adverse events.

### Mortality

**Fig. 4:**
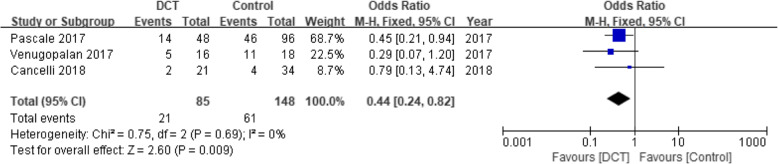
Forest plot of mortality for patients with CRE infection

Three studies consisting of 233 patients had reported the mortality with 30 ~ 60 days of follow-up visit. Compared with the control groups, DCT showed a lower mortality in patients with CRE infection (OR = 0.44, 95% CI = 0.24–0.82, *P =* 0.009) (Fig. 4).

### Summary of case series or case reports

A total of 18 case series or case reports composed of 90 patients were assessed. DCT regimens mainly consisted of ertapenem+meropenem and ertapenem+doripenem. Infection types included pneumonia, bloodstream infection, urinary tract infection, intra-abdominal infection, skin infection, surgical site infection and so on. The number of patients with bloodstream infection was the highest (31 patients, 34.4%). Followed by urinary tract infection (26 patients, 28.9%). The infections were primarily caused by MDR *K. pneumoniae*. Among 90 patients, clinical response was reported in 59 (65.6%) patients, while microbiological response was found in 63 (70%) patients. In patients with bloodstream infection, clinical and microbiological responses were 16/31 (51.6%) and 20/31 (64.5%), respectively. While both clinical and microbiological responses were 21/26 (80.8%) in patients with urinary tract infection. Nine cases of adverse events were reported, including seizures (three cases), aseptic meningitis (two cases), generalized rash (one case), eosinophilia (one case), nausea (one case) and hypernatremia (one case). The number of patients demised was 22 (24.4%), and 10 cases of them were reported to decease in spite of clinical or microbiological improvement.

## Discussion

In 2011, Bulik and Nicolau [55] first proposed the idea of DCT against KPC-producing *K. pneumoniae* by in vitro and mouse thigh infection model experiments. In 2013, Giamarellou et al. [26] reported that DCT successfully cured three patients with bloodstream infection and urinary tract infection caused by KPC-producing *K. pneumoniae*. Since then, DCT had been increasingly reported in clinical studies, no matter case series or case reports, cohort or case-control studies. Unfortunately, there were no RCTs available so far. To the best of our knowledge, our research was the first systematic review and meta-analysis of DCT to date, though two reviews had reported parts of DCT-treated patients with CRE infection. Our study contained three cohort or case-control studies consisting of 235 patients with CRE infection and 18 case series or case reports consisting of 90 patients. Most patients suffered from critical infections. The infection types mainly included pneumonia, bloodstream infection and urinary tract infection. Generally, the patients tolerated DCT regimens well. Only three case series had reported nine cases of adverse events, mainly including seizures and meningitis. Moreover, none of the adverse events led to interruption of treatment. Our meta-analysis demonstrated that though no obvious advantages in clinical and microbiological responses were noticed, the mortality in DCT regimens was lower compared with the control groups for CRE infection. In case series or case reports, ertapenem-containing regimens were the main pattern of DCT, which were applied to complicated severe infections caused by MDR Gram-negative bacteria. Our result was consistent with previous published reviews of DCT for the treatment of carbapenemase-producing *K. pneumoniae* caused critical infections [56] or CRE caused bloodstream infection [57] which both suggested that DCT regimens might be an effective and safe strategy to treat carbapenemase-producing *K. pneumoniae* or CRE. Moreover, White et al. [57] also revealed DCT exhibited lower mortality in the treatment of CRE bloodstream infection compared with polymyxin-based regimens. Oliva A et al. [58] compared DCT + colistin with DCT for the treatment of 32 patients with multiple infections caused by carbapenem-resistant *K. pneumoniae*. The result did not support that DCT + colistin was superior to DCT alone with similar clinical response and mortality in both groups.

DCT regimens have been proven to be effective in many in vitro and animal studies. In vitro studies [59, 60] have confirmed the synergistic effects of DCT regimens against carbapenemase-producing *K. pneumoniae*. Another in vitro study [61] has evaluated the synergistic activity of 10 double or triple combination regimens based on meropenem against carbapenemase-producing *K. pneumoniae*. The results show that the combination of meropenem and ertapenem is the most effective strategy in double combination regimens. DCT against MDR Gram-negative bacterial infections has also been supported by animal model data. The combination of ertapenem and doripenem has been observed to statistically decrease the bacterial density compared with doripenem monotherapy in a mouse thigh model infected with carbapenem-resistant *K. pneumoniae* [55]. The combination of doripenem and ertapenem has also exhibited a greater efficacy than doripenem alone at 72 h for KPC-producing *K. pneumoniae* infection in a neutropenic murine model with thigh infection [62].

The synergistic effects of DCT regimens mainly focus on the mechanism in combination with ertapenem. Ertapenem is considered to be the most sensitive to KPC enzyme in carbapenem antibiotics [63]. It can be hypothesized that ertapenem has preferential affinity with KPC and can consume the carbapenemases [55]. When ertapenem is combined with another carbapenem antibiotic, KPC is decreased per unit time so that another carbapenem antibiotic is hydrolyzed less. Higher concentration of another carbapenem antibiotic kills KPC-producing *K. pneumoniae* better [26]. An alternative explanation is that during treatment, ertapenem decreases the initial inoculum density by acting as a suicide substrate, thereby permitting doripenem to express its successful activity against an already reduced and manageable inoculum [64]. However, an in vitro study [59] has indicated that in DCT, the imipenem-containing combinations show the most efficacy in the treatment for carbapenemase-producing *K. pneumoniae* infection, while ertapenem may not be the best option to inactivate carbapenemases. This may be related to particularly enhanced in vitro activity of imipenem-containing combinations, even imipenem at sub-inhibitory concentrations [65]. However, in vivo data on imipenem-containing DCT are limited, which may be attributed to the central nervous system toxicity of imipenem and short stability of intravenous preparation [56, 66]. Meanwhile, meropenem can synergistically exert antibacterial effects by binding to the bacterial target, especially if minimal inhibitory concentration (MIC) value of meropenem is ≤128 μg/ml [60]. Further investigation is required since the treatment mechanism of DCT has not been extensively explored.

Although all available clinical evidence was included, there were four limitations in our systematic review and meta-analysis. Firstly, the studies included in the meta-analysis were all cohort or case-control studies, with case series or case reports as a supplement. The three studies including patients with CRE infection were all retrospective. The grade of evidence was insufficient. Secondly, publication and selective bias might exist. Two of three studies were from Italy, increasing the risk of bias due to geographic reasons. Thirdly, all the included studies did not provide information of resistance changes of pathogens after DCT exposure. Therefore, we were unable to know if excessive carbapenem exposure would lead to greater carbapenem resistance. At last, none of the antibiotics in control groups involved novel beta-lactam/beta-lactamase inhibitors (such as ceftazidime/avibactam). It was hard to evaluate how DCT would exhibit compared with the novel antibiotics.

## Conclusions

Collectively, due to similar efficacy response and lower mortality, DCT could be used as an alternative therapeutic option in the treatment of MDR Gram-negative bacterial infections. More high-quality clinical trials were required to further address the efficacy, safety and risk of carbapenem resistance of DCT.

## Data Availability

The datasets used or analyzed during this study are available from the corresponding author on reasonable requests.

## Abbreviations

DCT: Double-carbapenem therapy
D: DCT group
C: The control group
PNA: Pneumonia
HAP: Hospital-acquired pneumonia
VAP: Ventilator-associated pneumonia
BSI: Bloodstream infection
SBSI: Secondary bloodstream infection
CRBSI: Catheter-related bloodstream infection
API: Aortic periprosthetic infection
EPI: Endovascular prosthesis infection
EVDI: External ventricular drainage infection
CVCI: Central venous catheter infection
UTI: Urinary tract infection
IAI: Intra-abdominal infection
SSTI: Skin and soft tissue infection
STI: Soft tissue infection
SSSI: Skin and skin structure infection
SSI: Surgical site infection
MSI: Multiple site infection
KP: *Klebsiella pneumoniae*
EC: *Escherichia coli*
CRE: Carbapenem-resistant Enterobacteriaceae
CPCRE: Carbapenemase producing carbapenem-resistant Enterobacteriaceae
CRKP: Carbapenem-resistant *K. pneumoniae*
CPKP: Carbapenemase-producing *K. pneumoniae*
KPC: *K. pneumoniae* carbapenemase
KPC-II: A type II carbapenemase against KPC-producers
KPC-III: A type III carbapenemase against KPC-producers
SME: *Serratia marcescens* enzyme
NMC: Non-metallo enzyme carbapenemase
IMI: Imipenem-hydrolyzing
GES: Guiana extended spectrum
MBL: Metallo-beta-lactamase
IMP: Imipenemase
VIM: Verona integron-encoded MBL
SPM: Sao Paulo MBL
GIM: German imipenemase
NDM: New Delhi MBL
OXA: Oxacillinase
MDR: Multidrug resistant
XDR: Extensively drug resistant
PDR: Pandrug resistant
MEM: Meropenem
ETP: Ertapenem
DOR: Doripenem
GEN: Gentamicin
CST: Colistin
TGC: Tigecycline
RIF: Rifampicin
AMG: Aminoglycosides
CAB: Carbapenem antibiotics
FLQ: Fluoroquinolones
CIP: Ciprofloxacin
AMK: Amikacin
FOF: Fosfomycin
LZD: Linezolid
PMB: Polymyxin B
DOX: Doxycycline
FCA: Fluconazole
AVC: Ceftazidime/avibactam
RCT: Randomized controlled trial
NOS: Newcastle-Ottawa Scale
MIC: Minimum inhibitory concentration
SD: Standard deviation
OR: Odds ratio
CI: Confidence interval
S: Sensitive
I: Intermediate
R: Resistant
